# Wiretapping into microbial interactions by single cell genomics

**DOI:** 10.3389/fmicb.2015.00258

**Published:** 2015-04-08

**Authors:** Ramunas Stepanauskas

**Affiliations:** Bigelow Laboratory for Ocean SciencesEast Boothbay, ME, USA

**Keywords:** single cell genomics, microbial interactions, horizontal gene exchange, microevolution, microbial dark matter

Over the past decade, single cell genomics (SCG) made a swift transition from science fiction to a handy new tool in the biologist's toolset. The power of this technology lies in its ability to retrieve information-rich genomic blueprints from the most fundamental units of biological organization—individual cells. This is particularly significant in the case of bacteria, archaea, and protists, where single cells constitute complete organisms. Such unicellular individuals comprise the vast majority of biological diversity and biomass of our planet, yet only a small fraction of microbial diversity has been discovered and studied. Together with other modern research tools, SCG has been increasingly instrumental in deciphering the genomic composition, metabolic potential and evolutionary histories of the “microbial dark matter.” While cultivation-free recovery of discrete genomes was impossible in 2004, by 2009 it became a routine procedure that is accessible to the broad research community through open-access SCG core facilities (e.g., scgc.bigelow.org). This rapid development has enabled genomic studies of many previously unexplored branches of the tree of life (Marcy et al., 2007; Rinke et al., 2013) and findings of hitherto unrecognized biogeochemical processes and ecological patterns (Swan et al., 2011, 2013; Mason et al., 2012), paving the way for a new wave of discovery in microbiology and biotechnology.

An exciting feature that sets SCG apart from other cultivation-independent technologies is the retrieval of sequences of all the DNA molecules in a cell, in this way providing evidence for their physical co-occurrence (or absence) in the analyzed cell or consortia of multiple cells. Such molecules may include multiple chromosomes and plasmids of the host organism; genomes of organelles, symbionts, viruses, and other infecting agents and prey items; and naturally transformed DNA (Figure 1). The ability to collect this type of information offers a major, but still underutilized opportunity to microbiology. Infections, symbioses, phagotrophy, horizontal gene transfer, formation of consortia, and other interactions among unicellular, uncultured organisms and extracellular genetic elements can now be analyzed directly, in their natural environment. Such interactions are thought to be of paramount importance to the functioning of oceans, soils, macroorganismal (including human) biomes and other microbially-dominated ecosystems, although their *in situ* studies have been severely hampered by methodological difficulties.

**Figure 1:**
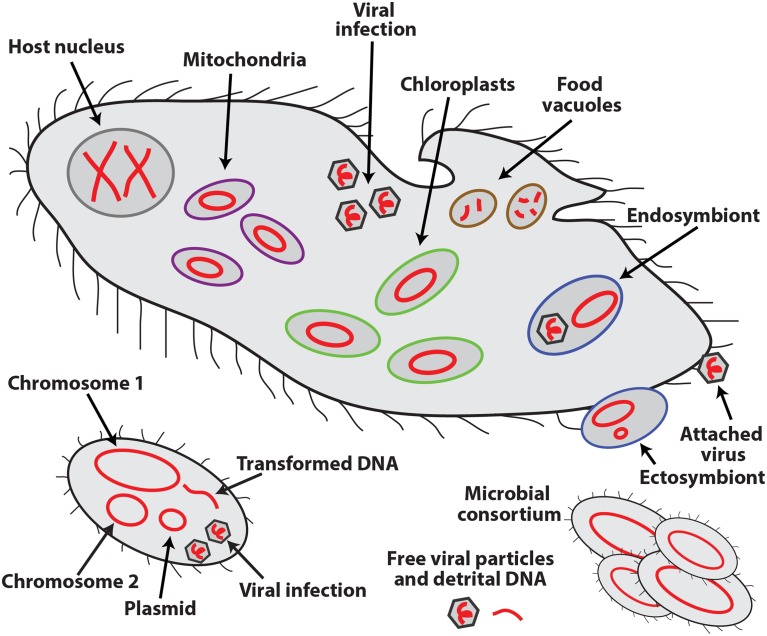
**Schematic representation of the various types of DNA molecules (in red) and their occurrence inside and outside of eukaryotic (top) and bacterial (bottom) cells**.

In one of the first applications of SCG on eukaryotes, multiple cells of the candidate phylum Picozoa (formerly Picobilliphyta) were found to contain fragments of bacterial and viral DNA, while no genes involved in photosynthesis were identified (Yoon et al., 2011). This provided the first evidence that Picozoa are phagotrophs, correcting prior suggestions of them being photosynthetic. The same SCG study also retrieved a complete genome of a novel nanovirus from an infected Picozoa cell. A phylogenetically broader screen of uncultured marine protists provided evidence for several novel symbiotic and phagotrophic interactions (Martinez-Garcia et al., 2012). More recently, SCG of planktonic bacteria from a model oxygen minimum zone revealed genomes and the spatiotemporal distribution of 69 novel phages infecting SUP05, an abundant but yet uncultured lineage of Gammaproteobacteria (Roux et al., 2014). Another SCG study, focused on surface ocean bacterioplankton, obtained genomes of the first known viruses of Thaumarchaeota, Marinimicrobia, Verrucomicrobia, and other ubiquitous, uncultured taxonomic groups of marine bacterioplankton (Labonte et al., in press). Using an innovative combination of SCG, metagenomics, and microarray hybridization, a virus infecting the candidate division Nanohaloarchaeota was identified (Martínez-García et al., 2014). It is likely that similar techniques will be increasingly utilized to untangle the complex inter-taxa interactions in “microbial jungles” of oceans, soils and other environments.

SCG is also becoming progressively instrumental in studies of microbial horizontal gene exchange (sexual interactions) and other microevolutionary processes in the environment. Microbial genomics came a long way since the first sequencing of a complete prokaryote genome, *Haemophilus influenzae* Rd, in 1995 (Fleischmann et al., 1995). As of January 2015, over 30,000 genomes of bacteria and archaea have been deposited in public databases, at an accelerating rate. While this is a truly revolutionary achievement for microbiology, it is important to acknowledge that we are still only scratching the surface of the full genetic complexity and the underlying evolutionary processes in many environmental microbial communities. To put current sequencing efforts in perspective, roughly 30,000 genomes of bacteria and archaea are present in each 30 μL of ocean water or 30 μg of a fertile soil (the same 30 μL water/30 μg soil also contain ~300,000 viruses, a few eukaryotes and a large amount of detrital DNA). The total number of microbial cells on the planet is in the order of 10^30^ (Whitman et al., 1998), which collectively encode at least 10^30^ Mbp of genetic information. An important fundamental question in microbiology is the extent and underlying mechanisms of genome variation among cells that share highly similar small subunit rRNA genes, i.e., belong to the same phylotype. Already the earliest comparative genomics studies revealed massive differences in gene content among members of the same, operationally defined bacterial species (Welch et al., 2002). Subsequent studies confirmed that substantial genome content variation among individuals, indicative of extensive horizontal gene exchange, is the rule rather than the exception in natural microbial populations (Ochman et al., 2000; Papke et al., 2007; Shapiro et al., 2012), with likely major implications to their resilience and adaptability to new conditions, such as climate change or exposure to antibiotics. From a technical perspective, the limited clonality of many natural microbial populations makes it difficult to assemble discrete genomes from metagenomic reads, and to unambiguously interpret such assemblies (Rusch et al., 2007; Hess et al., 2011). Our current understanding remains rudimentary when it comes to the extent of genetic variability within most microbial populations, specific evolutionary and ecological processes that govern this variability, and rates of these evolutionary processes. By eliminating the need for arbitrary taxonomic binning of omics data, SCG is well-suited to enable a breakthrough in studies of microbial microevolution, and first publications in this area have already provided valuable insights (Engel et al., 2014; Kashtan et al., 2014). These pilot studies suggest that each cell in a natural microbial population may have a unique subset of mutations, horizontally acquired and recombined genes, mobile genetic elements, and other genetic features. Instead of viewing this natural complexity as a nuisance, SCG enables “forensic DNA investigations” of individual microbial cells in the environment, opening a new window into their life histories.

In order to obtain statistically representative samples of members of natural microbial assemblages and their intra- and inter-species interactions, the scalability of SCG will be of key importance. In the past 5 years, the scale of SCG projects grew from single genomes (Marcy et al., 2007; Woyke et al., 2009) to 10 s and 100 s of genomes (Rinke et al., 2013; Swan et al., 2013; Kashtan et al., 2014), and further technology improvements are well-underway. Increasingly sophisticated research applications will also drive improvements in SCG data quality, such as better genome recovery and reduced frequency of assembly errors. Like any technology, SCG is at its best when combined with other research tools, in order to most effectively address transformative science questions. There is little doubt that SCG will be increasingly utilized in diverse microbial studies, complementing cultivation-based, community omics, biogeochemical, and other research approaches.

## Conflict of interest statement

The author declares that the research was conducted in the absence of any commercial or financial relationships that could be construed as a potential conflict of interest.
